# Evaluation of the Current Knowledge About Bacterial Endocarditis Prevention Among General Dentists in the City of Santo Domingo, Dominican Republic

**DOI:** 10.3389/fpubh.2020.585332

**Published:** 2020-11-24

**Authors:** Juan Manuel Aragoneses, Javier Aragoneses, Vanessa Arlette Brugal, Juan Algar, Ana Suarez

**Affiliations:** ^1^Department of Dental Research, Federico Henriquez y Carvajal University, Santo Domingo, Dominican Republic; ^2^Department of Dentistry, Federico Henriquez y Carvajal University, Santo Domingo, Dominican Republic; ^3^Department of Pediatric Dentistry and Orthopedics, School of Dentistry, Universidad Autónoma de Santo Domingo, Santo Domingo, Dominican Republic; ^4^Department of Clinical Dentistry, School of Biomedical Sciences, Universidad Europea de Madrid, Madrid, Spain; ^5^Department of Preclinical Dentistry, School of Biomedical Sciences, Universidad Europea de Madrid, Madrid, Spain

**Keywords:** bacterial endocarditis, preoperative antibiotic prophylaxis, amoxicillin, antibiotics, Dominican Republic

## Abstract

Infective endocarditis (IE) is a life-threatening disease caused by bacterial adherence to the lining of the heart and heart valve, and it can be caused by bacterial contamination of the bloodstream during invasive dental procedures. The American Heart Association (AHA) recommended guidelines for antibiotic prophylaxis in 2008 before invasive dental procedures; however, in the Dominican Republic, no official guidelines or regulations on this topic have been yet established. This study aimed to evaluate the current knowledge about bacterial endocarditis prevention among dentists in Santo Domingo. The study participants were dentists who attended a conference organized by Universidad Federico Henríquez y Carvajal (*n* = 95), of which 74 responded to the questionnaire survey. Seventy-eight percentage of the participants responded that an indication of antibiotics is recommended in cases of prophylaxis for IE. The prescription of antibiotics was applied to patients with prosthetic valves (78.4%), presented a history of previous IE (77%) among others. Among all the interventions in which the respondents would prescribe antibiotics, tooth extraction (70.7%) was the most frequent. Amoxicillin was the preferred drug choice (63.5%) and clindamycin was the antibiotic of choice in allergic patients (55.4%). Even though the choice of antibiotics were according to AHA guidelines (2008), majority of the dentists (58.82 and 55.4%) were not aware of the correct dosage and timing of administration of azithromycin and clindamycin in drugs in patients allergic to penicillin.

## Introduction

Infective endocarditis (IE) is a life-threatening disease that is caused by bacterial adherence to the lining of the heart and the valves of the heart (1). The morbidity and mortality rates in the affected patients can be as high as 80% (2). The survival of the patients with repeated infections of this disease has been reduced to 60% (3). A 10-year review of cases reported that the incidence of this disease was 0.36 cases per million population and there was about 16% median in-hospital mortality. The disease is either acquired, congenital, or due to cardiac defects, however, it has also been shown to occur during dental procedures where the existing data is still controversial (1, 4, 5). During tissue injury, a variety of microorganisms present in the oral cavity can enter into the bloodstream. Invasive dental procedures like root canal treatment, extractions, and periodontal procedures can result in increased infections in the heart, especially in patients already suffering from heart disease (2). Hence, some authors recommend antibiotic prophylaxis for patients suffering from a heart condition that may catalyze in IE (1, 2).

The most commonly used guidelines for antibiotic prophylaxis have been proposed by the American Heart Association (AHA) in 2008 and that has been approved by American Dental Association (ADA) and endorsed by the Canadian Dental Association (2, 6). The guidelines state that antibiotic prophylaxis is required in dental procedures where there is a manipulation of gingival tissues, periapical region, or perforation of oral mucosa that has been performed in patients with specified cardiac conditions (ex: prior episode of IE, prosthetic valve) (2). According to the guidelines, the recommended antibiotic regimen is Amoxicillin for adults (2 g) and children (50 mg/kg), at least 30 min before the start of the dental procedure. If adults were unable to consume oral medication, 2 g Ampicillin, or 1 g Cefazolin or Ceftriaxone by intramuscular (IM) or intravenous (IV) administration has been recommended. Drugs like Azithromycin or Clarithromycin have been suggested in patients who were allergic to penicillin (2).

There was a stark contrast to the guidelines published by the National Institute for Health and Clinical Excellence (NICE) in 2008 (7). It stated that patients undergoing interventional procedures that involve the dental, upper and lower respiratory tract, genitourinary tract, or upper and lower gastrointestinal tract need not be offered antibiotic prophylaxis against infective endocarditis and patients with pre-existing cardiac conditions should be considered as high-risk (7). But, a study conducted by Dayer et al., between the years 2000 and 2013 showed that there was a higher incidence of cases with IE following the publication of NICE guidelines (8). However, the recommendations have been upheld by NICE during its review in 2015 and it had added that chlorhexidine mouthwash cannot be given to patients as a mode of prophylaxis for patients prone to IE (9). In 2016, the institute amended its recommendation stating that when the individuals were considered to be at high risk for IE or when patients preferentially expressed the need for antibiotic prophylaxis, then the prophylactic regimen may be appropriate in such cases (10).

Despite the many modifications in guidelines proposed by various organizations, there is still a dearth of evidence regarding the effectiveness of antibiotic prophylaxis against infective endocarditis (11, 12). There has been a cloud of controversy regarding the NICE guidelines and this may be because all these recommendations were based on observational cohort studies rather than randomized controlled clinical trials (11, 13, 14). In the US, a study conducted by Lockhart et al., showed that there was a high acceptance of 2007 (>75%) AHA guidelines when a survey was conducted among 901 dentists in routine clinical practice (15). Another study conducted by Elad et al., in 2010 observed that the acceptance of AHA guidelines remained moderate to high among patients in Israel (16). The knowledge and acceptance of these guidelines were found to be significantly high among Israeli dentists (17). But, there has been a paucity of evidence regarding the knowledge and awareness of pre-operative antibiotic prophylaxis among dentists in the Dominican Republic. With due consideration to the lack of guidelines proposed by any official organization in the country, the aim of this cross-sectional study was to evaluate the current knowledge and awareness about the prevention of IE among dentists in the city of Santo Domingo.

## Materials and Methods

The research protocol was approved by Universidad Federico Henríquez y Carvajal, Ethics Committee (2/04/2017) and it followed the Helsinki declaration. A survey was carried out among dentists (*n* = 95) who attended a conference organized by Universidad Federico Henríquez y Carvajal in 2017 for improvement in teaching methodologies. The conference was attended by Professors from the University and many dentists from the city of Santo Domingo. After reading the summary of the study protocol, and informed consent was obtained from the study participants who were willing to participate. The attendants who refused to participate or provided incomplete surveys were excluded from the study. The questionnaire was delivered and collected by one of the researchers in this study.

### Questionnaire

The questionnaire comprised two sections (Appendix) where the initial three questions probed the demographic information about the dentists such as age, sex, and professional experience (in years). There were two questions on the knowledge regarding antibiotic prophylaxis depending on the source i.e., either by classroom teaching in the undergraduate degree or by reading research articles among dentists and the score was assessed based on a scale from 1 to 10. The remainder of the questions focused on the knowledge and awareness among dentists about IE, the cardiac conditions warranting antibiotic prophylaxis, the dental procedures that require prophylaxis, and the commonly used antibiotics for the prophylactic regimen. It also assessed the dentists' awareness of drug usage in allergic and non-allergic patients. The question on the awareness of cardiac conditions that require prophylaxis according to AHA guidelines consisted of the following 16 conditions: congenital heart disease, prosthetic valve, previous history of IE, stent, ischemic heart disease, atrial septal defects, ventricular septal defect, rheumatic heart disease, untreated cyanotic heart disease, heart bypass surgery, pacemaker insertion, mitral valve prolapse or regurgitation, physiological heart murmur, history of cardiac surgery, heart transplant, and heart transplant patients with valvular disease.

The awareness among dentists on the dental procedures that warrant antibiotic prophylaxis was assessed by proposing a multitude of different procedures (*n* = 25) from various specialties in dentistry: Infiltrative local anesthesia, inferior alveolar nerve block, intraligamentary anesthesia, tooth extraction, replantation of teeth, incision and drainage of the abscess, scaling using an ultrasonic scaler, periodontal probing, scaling and root planning, periodontal surgery, dental implant surgery, bone grafting, sinus lift, suture removal, Implant surgery (second stage), biopsy, endodontic therapy, apicoectomy, dental isolation using clamps, dental impression, orthodontic band placement, gingival retraction using cords, tooth carving that includes gingival bleeding, matrix band, and wedge placement, and removal of subgingival caries. The last section of the questionnaire focused on the type of antibiotic drug, timing, and dosage to be used in allergic and non-allergic patients for the prevention of IE.

### Statistical Analysis

The data were entered into Microsoft Excel workbook and exported into SPSS v21.0 (IBM, SPSS Inc, Chicago Ill., USA). The data has been expressed as frequency, percentages, median, and interquartile range. Skewed data were compared using Mann Whitney *U*-test. A *p*-value of less than 0.05 was considered statistically significant.

## Results

### General Characteristics

Among 95 dentists who attended the conference, only 74 participants were included in the questionnaire study. The demographic characteristics of the study population have been depicted in Table 1. About 37.84% of the participants were between 35–44 and 45–54 years' age group and when combined, they made up 75% of the study population. The gender ratio (male: female) in the study population was 0.57:1, with considerably more female dentists, participated in the study. More than half of the study population (54%) had professional clinical experience between 12 and 22 years.

**Table 1:** Demographic characteristics of the study population.

**Variables**	**Frequency**	**Percentages**
**Age (years)**		
25–34	11	14.86
35–44	28	37.84
45–54	28	37.84
55–64	7	9.46
**Sex**		
Male	27	36.49
Female	47	63.51
**Professional experience (years)**		
0–11	16	21.62
12–22	40	54.05
23–33	18	24.32

### Knowledge Score

The median knowledge score acquired during classroom study was significantly lower than the score obtained through reading research articles [7.0 (6.0, 8.0) vs. 8.0 (7.0, 9.0)] and it showed very high statistical significance with *p* < 0.0001.

### Indication for Antibiotic Prophylaxis for IE

The dentists were questioned on the cardiac conditions that indicate antibiotic prophylaxis based on the current guidelines. The majority of the participants (78.4%) mentioned prosthetic valve, followed by the previous infection of IE (77%), rheumatic heart disease (74.3%), congenital heart disease (67.6%), and atrial septal defects (63.5%). The least percentage of responses were for conditions like physiological heart murmur (5.4%), pacemaker insertion (12.2%), and mitral valve prolapse or regurgitation (23%) (Table 2).

**Table 2:** Assessment of awareness among dentists regarding the indication (cardiac condition) of antibiotic prophylaxis for prevention of IE.

**Cardiac conditions**	**Frequency**	**Percentages**
Prosthetic valve	58	78.4
Previous infectious endocarditis	57	77.0
Rheumatic heart disease	55	74.3
Congenital heart disease	50	67.6
Atrial septal defects	47	63.5
Heart transplant patients with heart valve disease	47	63.5
Ventricular septal defect	45	60.8
Untreated cyanotic heart disease	43	58.1
Stent	43	58.1
Ischemic heart disease	37	50.0
History of cardiac surgery	36	48.6
Heart bypass surgery	30	40.5
Heart transplant	25	33.8
Mitral valve prolapse or regurgitation	17	23.0
Pacemaker insertion	9	12.2
Physiological heart murmur	4	5.4

### Dental Procedures for Prescribing Antibiotics for the Prevention of IE

The majority of the respondents stated dental procedures like tooth extraction (70.7%), abscess drainage (70.7%), inferior alveolar nerve block (68.9%), tooth replantation (68.9%), bone grafting (62.2%), and dental implant surgery with or without sinus lift (60.8%) require an antibiotic prescription for prevention of IE. The dental procedures that received the least positive response were for matrix band and wedge placement (5.4%), gingival retraction using cords (6.8%), a dental impression (6.8%), and subgingival caries removal (8.1%) (Table 3).

**Table 3:** Assessment of awareness among dentists for different dental procedures that indicate antibiotic prophylaxis to prevent IE.

**Dental procedures**	**Frequency**	**Percentages**
Tooth extraction	52	70.7
Incision to drain an abscess	52	70.7
Inferior alveolar nerve block	51	68.9
Replantation of teeth	51	68.9
Bone grafting	46	62.2
Dental implant surgery	45	60.8
Sinus lift	45	60.8
Apicoectomy	44	59.5
Scaling and root planing	42	56.8
Periodontal surgery	42	56.8
2nd implant surgery	42	56.8
Biopsy	42	56.8
Dental cleanings using ultrasonic scalers	36	48.6
tooth carving that include gingival bleeding	36	48.6
Periodontal probing	31	41.9
Infiltrative local anesthesia	28	37.8
Intraligamentary anesthesia	28	37.8
Endodontics therapy	24	32.4
Suture removal	21	28.4
Dental isolation clamps placement	20	27.0
Orthodontic bands placement	10	13.5
Removal of subgingival caries	6	8.1
Dental impression	5	6.8
gingival retraction with retraction cords	5	6.8
Matrix band and wedges placement	4	5.4

### Choice of Antibiotics for IE

Amoxicillin was the most preferred antibiotic among dentists with over 63.5% of the study population warranting its usage for the prevention of IE in non-allergic patients when performing dental procedures. It was followed by amoxicillin+clavulanic acid (5.4%), clindamycin (2.7%), and azithromycin (1.35%) was the least preferred antibiotic among the dentists (Table 4). In allergic patients, the preferred antibiotic and dosage was azithromycin (500 mg, 2 h before the procedure) followed by clindamycin (300 mg, 1 day prior to procedure), and clindamycin (600 mg, 1 h before the procedure). The least likely antibiotic dosing and timing was clindamycin (300 mg, 1 h before the procedure) and azithromycin (500 mg immediately before the procedure) (Table 5).

**Table 4:** Choice of antibiotics in non-allergic patients for the prevention of IE among the dentists.

**Antibiotic drug**	**Frequency**	**Percentages**
Amoxicillin	47	63.5
Azithromycin	1	1.35
Clindamycin	2	2.70
Amoxicillin + clavulanic acid	4	5.40

**Table 5:** Choice of antibiotics in allergic patients for the prevention of IE among the dentists.

**Antibiotic drug**	**Frequency**	**Percentages**
**Azithromycin**	17	22.97
500 mg 1 day before 500 mg 2 h before 500 mg 1 h before 500 mg immediately before	10 3 3 1	58.82 17.65 17.65 5.88
**Clindamycin**	41	55.40
300 mg 1 day before 300 mg 2 h before 300 mg 1 h before 300 mg immediately before 600 mg 1 day before 600 mg 2 h before 600 mg 1 h before 600 mg immediately before	3 2 1 2 2 10 14 7	7.32 4.88 2.44 4.88 4.88 24.39 34.15 17.07

## Discussion

In this study, the current knowledge and awareness regarding antibiotic prophylaxis for the prevention of IE among general dentists (*n* = 74) in Santo Domingo, Dominican Republic was evaluated using a questionnaire-based survey. The majority of the participants were between 35 and 54 years of age with about half of the study population having 12–22 years of professional experience. At the time of the study, there was no official census of the number of dentists working in the Dominican Republic, hence this cross-sectional survey included data from dentists with a wider age range and experience distribution.

About 78% of the study population responded that antibiotic prophylaxis was warranted for the prevention of IE. In a study conducted by Al-Fouzan et al., reported that the knowledge level regarding the prevention of IE was around 52.2% and variations existed between specialists, general dentists, and dental interns (18). A systematic review that included 40 articles was performed by Cummins et al., and it showed that the general knowledge of the guidelines ranged between 1.9 and 100% and there was a variation between dental students and qualified dentists (19). Although this study was conducted only among general dentists, the knowledge level was within the range mentioned in the previous studies and it may be reasonable to suppose that majority of the dentists in the Dominican Republic had the knowledge about antibiotic guidelines for the prevention of IE.

A study by Boyle et al. reported that 56% of dental practitioners in Ireland were aware of the guidelines toward antibiotic prophylactic regimen (20). In another study, the dentists' knowledge about antibiotic prophylaxis for the prevention of IE was comparatively low with <50% of the dentists showing adequate level (21). In our study, the median knowledge score about antibiotic prophylaxis was 7.0. A study by Kumar et al., conducted among 100 undergraduate dental students from a dental school in India reported that 73% of the student population were aware of the guidelines toward antibiotic usage for IE prevention (22). The observation was in stark contrast to the findings in this study which reported that the median knowledge scores among dentists acquired through reading research articles were higher when compared to classroom teaching. This may be due to differences in undergraduate dental curriculum followed in different countries and this finding warrants attention toward more focused research on this topic.

In this study, the dentists were surveyed about the different cardiac conditions that might warrant antibiotic prophylaxis for the prevention of IE. More than half of the study population responded to prosthetic cardiac valves as a primary indication for antibiotic prophylaxis toward the prevention of IE. Prosthetic valves remain to be one of the major risk factors for the development of IE (23). In a study conducted by Hashemipour et al., among dentists who attended the 47th dental international congress on the prophylactic regimen for prevention of IE, about 95% of the respondents positively indicated that antibiotic prophylaxis was necessary for patients with prosthetic heart valves (21). The British Society for Antimicrobial Chemotherapy has recommended antibiotic prophylaxis before dental procedures exclusively for patients with a history of previous IE infection or with cardiac valve replacement (24). Another study conducted by Ryalat et al., reported that 87% of Jordanian dentists recommended antibiotic prophylaxis in patients with prosthetic devices (25). In this study, the vast majority of the study population was aware of the general cardiac indications for antibiotic prophylaxis toward the prevention of IE.

A study conducted by Cloitre et al., reported that 98% of the respondents recommended antibiotic prophylaxis for tooth extraction and bone tissue surgery, 97% for soft tissue surgery, and 85% for endodontic therapy of vital monoradicular teeth (26). Ryalat et al., observed that most dentists felt that dental extractions required prophylaxis (94.5%), followed by periodontal surgery (88.2%), but only 45.7% thought that endodontic therapy might require prophylaxis. In this study, similar results were obtained with 70.7% of the respondents recommending prophylaxis for tooth extractions and abscess drainage, 62.2% for bone grafting procedures, and 56.8% for periodontal surgery; however, only 32.4% of dentists recommended prophylaxis for endodontic therapy.

The study conducted by Ryalat et al., observed that amoxicillin was the preferred choice of the antibiotic drug among dentists to prevent IE, followed by clindamycin (17.3%), and a minority of the dental population preferred metronidazole, lincomycin, gentamycin, or clarithromycin (25). It was also the drug of choice among Nigerian dentist with 89% preferring it, however, Japanese dentists preferred cephams followed by penicillin drugs (27, 28). In this study, amoxicillin remained the preferred antibiotic drug among dentists with 63.5% warranting its usage for the prevention of IE. In allergic patients, AHA (2008) recommended Cephalexin, Clindamycin, Azithromycin, or Clarithromycin antibiotic prophylaxis for the prevention of IE (2). In this study, more than half of the study population (58.82%) recommended azithromycin 2 h prior to the dental procedure. Only three dentists prescribed Azithromycin 500 mg 1 h before the procedure, whereas one dentist reported use of 500 mg Azithromycin immediately before the procedure. The standard guidelines recommend a single dose of 500 mg Azithromycin 30–60 min prior to a dental procedure (2). Clindamycin has been recommended as a single dose of 600 mg, 30–60 min before a dental procedure, while in this study, 33 dentists were aware of the specific dosage but not about the timing. It is reasonable to suppose that the findings from this study observed that the dentists were not clear about the dosage and timing of the antibiotic prophylaxis to be implemented prior to a dental procedure to prevent IE.

The AHA guidelines state that antibiotic prophylaxis is warranted for high-risk individuals prior to certain dental procedures. The dentists in this study were aware of the indications and dental procedures that warrant prophylactic antibiotic usage, but they were not sure of the timing and dosage of the antibiotics. However, the limitation of this study includes the small sample size, so a generalized conclusion cannot be drawn from the results. This is the first study to assess the knowledge and awareness of antibiotic prophylaxis practices to prevent IE in the dental population in the Dominican Republic. It is to be noted that poor knowledge of the guidelines can result in misuse of antibiotics and antibiotic resistance and this study emphasized the need for continuing dental education, the inclusion of standard guidelines in the undergraduate curriculum, and formal issuance of guidelines from regulatory bodies in the Dominican Republic.

## Data Availability Statement

The raw data supporting the conclusions of this article will be made available by the authors, without undue reservation.

## Ethics Statement

The studies involving human participants were reviewed and approved by Federico Henriquez y Carvajal University. The patients/participants provided their written informed consent to participate in this study.

## Author Contributions

JMA, JAr, and AS designed the study, participated in its coordination, and drafted the manuscript. VB helped draft the manuscript. JAr and JAl participated in data analysis and interpretation. All authors read and approved the final manuscript.

## Conflict of Interest

The authors declare that the research was conducted in the absence of any commercial or financial relationships that could be construed as a potential conflict of interest.
