# Supplementary material for: Evaluation of the Current Knowledge About Bacterial Endocarditis Prevention Among General Dentists in the City of Santo Domingo, Dominican Republic
Source: Front Public Health. 2020 Nov 24;8:585332. doi: 10.3389/fpubh.2020.585332 (PMC7732663; doi:10.3389/fpubh.2020.585332)
Supplement: Supplementary file 1 [file Data_Sheet_1.docx]

INFORMED CONSENT TO PARTICIPATE IN THE STUDY:

I have been informed of the objectives of the study, and I have been told what my participation consists of. I understand that the information I provide over the course of this research is strictly confidential and will not be used for any purpose other than that of the present study without my consent. I have been informed that I can ask questions about the project at any time and that I can withdraw from it when I so choose, without this having any negative repercussions on me.

SURVEY QUESTIONS:

• Age

• Sex

• Years of professional practice.

• On a scale of 1 to 10, according to you, how much knowledge did you acquire during your undergraduate degree on the subject of antibiotic therapy?

• On a scale of 1 to 10, according to you, how much knowledge did you acquire about antibiotic therapy by reading?

• Do you indicate antibiotics in cases of prophylaxis for bacterial endocarditis?

• In which cases would you prescribe an antibiotic for the prevention of bacterial endocarditis? Patients with…

| Congenital heart disease |
| --- |
| Prosthetic device |
| Previous infectious endocarditis |
| Stent |
| Ischemic heart disease |
| Atrial septal defects |
| Ventricular septal defect |
| Rheumatic heart disease |
| Untreated Cyanotic Heart Disease |
| Heart bypass surgery |
| Pacemaker insertion |
| Mitral valve prolapse or regurgitation |
| Physiological heart murmur |
| History of cardayc surgery |
| Heart transplant |
| Heart transplant patients with heart valve disease |

• In which of the following procedures do you prescribe antibiotics as prophylaxis to prevent bacterial endocarditis?

| Infiltrative local anesthesia |
| --- |
| Inferior Alveolar Nerve Block |
| Intraligamentary anesthesia |
| Tooth extraction |
| Replantation of teeth |
| Incision to drain an abscess |
| Dental Cleanings Using Ultrasonic Scalers |
| Periodontal probing |
| Scaling and root planing |
| Periodontal surgery |
| Dental implant surgery |
| Bone grafting |
| Sinus lift |
| Suture removal |
| 2nd implant surgery |
| Biopsy |
| Endodontics therapy |
| Apicoectomy |
| Dental isolation clamps placement |
| Orthodontic bands placement |
| Dental impression |
| Gingival retraction with retraction cords |
| Tooth carving that include gingival bleeding |
| Matrix band and wedges placement |
| Removal of subgingival caries |

• Which of the following antibiotics do you usually prescribe for bacterial endocarditis?

| Amoxicillin 500mg 1 day before |
| --- |
| Amoxicillin 500mg 2 hours before |
| Amoxicillin 500mg 1 hour before |
| Amoxicillin 500mg inmediately before |
| Amoxicillin 1000mg 1 day before |
| Amoxicillin 1000mg 2 hours before |
| Amoxicillin 1000mg 1 hour before |
| Amoxicillin 1000mg inmediately before |
| Amoxicillin 2000mg 1 day before |
| Amoxicillin 2000mg 2 hours before |
| Amoxicillin 2000mg 1 hour before |
| Amoxicillin 2000mg inmediately before |
| Azithromycin 500mg 1 day before |
| Azithromycin 500mg 2 hours before |
| Azithromycin 500mg 1 hour before |
| Azithromycin 500mg inmediately before |
| Amoxicillin875 mg + clavulamic acid 1 day before |
| Amoxicillin 875 mg + clavulamic acid 2 hours before |
| Amoxicillin 875 mg + clavulamic acid 1 hour before |
| Amoxicillin 875 mg + clavulamic acid inmedaytely before |
| Azithromycin 500mg 1 day before |
| Azithromycin 500mg 2 hours before |
| Azithromycin 500mg 1 hour before |
| Azithromycin 500mg inmedatly before |
| Clindamycin 300mg 1 day before |
| Clindamycin 300mg 2 hours before |
| Clindamycin 300mg 1 hour before |
| Clindamycin 300mg inmediately before |
| Clindamycin 600mg 1 day before |
| Clindamycin 600mg 2 hours before |
| Clindamycin 600mg 1 hour before |
| Clindamycin 600mg inmediately before |
| Other |

• If your answer was “Other ”, please specify which antibiotic you use, in what dosage, how often and for how many days.

• IN ALLERGIC PATIENTS Which one does it prescribe?

| Azithromycin 500mg 1 day before |
| --- |
| Azithromycin 500mg 2 hours before |
| Azithromycin 500mg 1 hour before |
| Azithromycin 500mg inmedatly before |
| Clindamycin 300mg 1 day before |
| Clindamycin 300mg 2 hours before |
| Clindamycin 300mg 1 hour before |
| Clindamycin 300mg inmediately before |
| Clindamycin 600mg 1 day before |
| Clindamycin 600mg 2 hours before |
| Clindamycin 600mg 1 hour before |
| Clindamycin 600mg inmediately before |
| Other. |

• If your answer was “Other ”, please specify which antibiotic you use, in what dosage, how often and for how many days.
